# Supplementary material for: Alterations of lower- and higher-order aberrations after unilateral horizontal rectus muscle surgery in children with intermittent exotropia: A retrospective cross-sectional study
Source: PLoS One. 2022 Feb 17;17(2):e0264037. doi: 10.1371/journal.pone.0264037 (PMC8853472; doi:10.1371/journal.pone.0264037)
Supplement: S1 Table — LR: lateral rectus; MR: medial rectus; PD: prism diopter. (DOCX) [file pone.0264037.s001.docx]

**S1 Table. Classification of the extent of surgery by exodeviation prism diopter**

| **Exodeviation angle** | **Extent of surgery (LR recession/MR resection)** | **Number of patients** |
| --- | --- | --- |
| 20 PD | 5.0 mm/4.0 mm | 14 |
| 25 PD | 6.0 mm/5.0 mm | 37 |
| 30 PD | 7.0 mm/5.5 mm | 17 |
| 35 PD | 7.5 mm/6.0 mm | 10 |
| 40 PD | 8.0 mm/6.5 mm | 6 |
| 50 PD | 10.0 mm/7.0 mm | 1 |
| Total |  | 85 |

LR: lateral rectus; MR: medial rectus; PD: prism diopter
