# Supplementary material for: Alterations of lower- and higher-order aberrations after unilateral horizontal rectus muscle surgery in children with intermittent exotropia: A retrospective cross-sectional study
Source: PLoS One. 2022 Feb 17;17(2):e0264037. doi: 10.1371/journal.pone.0264037 (PMC8853472; doi:10.1371/journal.pone.0264037)
Supplement: S2 Table — IOP: intraocular pressure; LOA: lower-order aberrations; HOA: high-order aberrations. (DOCX) [file pone.0264037.s002.docx]

**S2 Table. Changes in the IOP, refraction, corneal power, LOAs, and HOAs compared to the preoperative baseline.**

| Variable | Postop 1 week | Postop 3 month |
| --- | --- | --- |
| IOP (mmHg) | 0.25 ± 0.42 | 0.16 ± 0.4 |
| Spherical (D) | -0.67 ± 0.10** | -0.49 ± 0.11** |
| Cylinder (D) | -0.75 (0.88)**^$^ | -0.48 (1.19)**^$^ |
| Axis (°) | 0.00 (70)**^$^ | 0.00 (42.75)*^$^ |
| Simulated Mean K (D) | 0.15 ± 0.05** | 0.07 (0.35) ^$^ |
| Simulated Flat K (D) | -0.31 ± 0.13* | -0.19 ± 0.13 |
| Axis 1 (°) | 0.00 (19.5) ^$^ | 1.00 (14) ^$^ |
| Simulated Steep K (D) | 0.51 ± 0.07** | 0.18 ± 0.05** |
| Axis 2 (°) | 0.67 ± 1.84 | 2.86 ± 1.59 |
| Simulated Astigmatism (D) | 0.68 (0.72)**^$^ | 0.25 (0.51)**^$^ |
| Axis 3 (°) | 0.00 (13.5) ^$^ | 1.00 (11) ^$^ |
| Ray Tracing Mean K (D) | 0.14 ± 0.06* | 0.83 ± 0.07** |
| Ray Tracing Flat K (D) | -0.22 ± 0.08** | 0.59 ± 0.08** |
| Axis 1 (°) | 1.00 (41.5) ^$^ | 2.00 (71) ^$^ |
| Ray Tracing Steep K (D) | 0.45 ± 0.08** | 1.05 ± 0.08** |
| Axis 2 (°) | 3.95 ± 2.40 | 2.12 ± 2.00 |
| Ray Tracing Astigmatism (D) | 0.62 (0.83)**^$^ | 0.29 (0.61)**^$^ |
| Axis 3 (°) | 3.95 ± 2.40 | 5.15 ± 1.93** |
| RMS (μm) | 0.65 (0.67)**^$^ | 0.24 ± 0.05** |
| 2nd Defocus (μm) | 0.69 ± 0.07** | -0.02 ± 0.04 |
| 2nd Oblique Astigmatism (μm) | 0.08 ± 0.07 | -0.03 ± 0.06 |
| 2nd Vertical Astigmatism (μm) | -0.37 ± 0.13** | -0.24 ± 0.09** |
| 3rd Vertical Coma (μm) | -0.05 ± 0.04 | 0.00 ± 0.02 |
| 3rd Horizontal Coma um | 0.02 ± 0.02 | 0.00 ± 0.02 |
| 3rd Oblique Trefoil (μm) | 0.09 ± 0.04* | 0.01 ± 0.02 |
| 3rd Horizontal Trefoil (μm) | 0.00 ± 0.03 | 0.02 ± 0.02 |
| 4th Spherical Aberration (μm) | -0.06 (0.13)**^$^ | 0.00 ± 0.01 |
| 4th Oblique Secondary Astigmatism (μm) | 0.00 ± 0.01 | -0.01 ± 0.01 |
| 4th Vertical Secondary Astigmatism (μm) | 0.04 (0.11)**^$^ | -0.02 ± 0.01* |
| 4th Oblique Quadrafoil (μm) | 0.01 ± 0.02 | 0.01 ± 0.02 |
| 4th Vertical Quadrafoil (μm) | -0.05 (0.23)*^$^ | 0.01 ± 0.02 |
| 2nd (sum) | 0.67 ± 0.09** | 0.26 ± 0.05** |
| 3rd (sum) | 0.08 (0.29)**^$^ | -0.01 ± 0.02 |
| 4th (sum) | 0.04 (0.17)**^$^ | 0.02 ± 0.01* |
| 5th (sum) | 0.03 (0.13)**^$^ | 0.00 (0.14) ^$^ |
| 6th (sum) | 0.02 (0.12)**^$^ | 0.01 (0.09) ^$^ |
| 7th (sum) | 0.01 (0.05)**^$^ | 0.00 (0.04) ^$^ |
| 8th (sum) | 0.01 (0.03)**^$^ | 0.00 (0.03) ^$^ |

IOP: intraocular pressure; K: keratometry; D: diopter; RMS: root mean square.

Normally distributed variables are expressed as mean ± standard error

Non-normally distributed variables are expressed as median (interquartile range)

p values are computed using a paired *t*-test or the Wilcoxon signed-rank test^$^ according to the normality of data distribution of the variables

p value* < 0.05, p value** < 0.01
